# Melanoma Detection in Pigmented Lesions ≤ 6 mm Selected for Excision After Dermoscopy in Routine Practice: A Retrospective Cross-Sectional Study

**DOI:** 10.3390/cancers18142183

**Published:** 2026-07-08

**Authors:** Vincenzo De Giorgi, Giovanni Cecchi, Virginia Marabini, Ginevra Gurioli, Gabriella Perillo, Federica Fazzari, Biancamaria Zuccaro

**Affiliations:** 1 Section of Dermatology, Department of Health Sciences, University of Florence, 50100 Florence, Italy; giovanni.cecchi1@unifi.it (G.C.); virginia.marabini@edu.unifi.it (V.M.); gabriella.perillo@unifi.it (G.P.); federica.fazzari@unifi.it (F.F.); biancamaria.zuccaro@unifi.it (B.Z.); 2 Cancer Research “Attilia Pofferi” Foundation, 51100 Pistoia, Italy; ginevra.gurioli@edu.unifi.it

**Keywords:** melanoma, dermoscopy, pigmented lesions, diagnostic yield, lesion diameter

## Abstract

**Background**: Dermoscopy has increased the excision of very small pigmented lesions in routine practice, yet the diagnostic efficiency of excising lesions ≤ 6 mm judged suspicious for melanoma remains incompletely defined. **Objectives**: The objectives of this study were to quantify melanoma detection yield and number needed to excise (NNE) for pigmented lesions ≤ 6 mm excised after clinical and dermoscopic suspicion of melanoma and to describe melanoma characteristics according to lesion diameter. **Methods**: We performed a retrospective observational cross-sectional study at a tertiary dermatologic oncology referral center within the Tuscany Regional Health Service, Florence, Italy. All pigmented lesions excised between 1 January 2022 and 31 December 2023 were screened. Consecutive lesions were included if they were excised because melanoma or suspected melanoma was the preoperative clinicodermoscopic diagnosis; lesions removed for benign or cosmetic indications were excluded. Primary outcomes were melanoma yield and number needed to excise, overall and stratified by clinical diameter. Multivariable logistic regression was performed to assess whether lesion diameter was independently associated with melanoma diagnosis after adjustment for age, sex, and anatomical site. **Results**: Among 2240 included excisions, 609 melanomas were diagnosed, corresponding to an overall yield of 27.2%. Lesions ≤ 6 mm accounted for 1331 excisions and yielded 175 melanomas, with a yield of 13.1% and a number needed to excise of 7.6. Lesions > 6 mm accounted for 909 excisions and yielded 434 melanomas, with a yield of 47.7% and a number needed to excise of 2.1. Thus, 86.9% of excised lesions ≤ 6 mm were benign on histopathology. Melanomas diagnosed in lesions ≤ 6 mm showed a more favorable histopathologic profile than larger melanomas. In multivariable analysis, lesions > 6 mm had significantly higher odds of melanoma than lesions ≤ 6 mm after adjustment for age, sex, and anatomical site (adjusted odds ratio 4.77, 95% confidence interval 3.81–5.97; *p* < 0.001). **Conclusions**: In routine practice, excision of dermoscopically suspicious pigmented lesions ≤ 6 mm has markedly lower melanoma yield and higher NNE than excision of larger lesions. These findings support risk-stratified management approaches and careful consideration of excision thresholds for very small lesions.

## Simple Summary

Small pigmented skin lesions are frequently removed when they appear suspicious during clinical and dermoscopic examination, but it is not always clear how often these very small lesions are actually melanoma. This study investigated pigmented lesions removed in routine clinical practice because melanoma was suspected, comparing lesions measuring 6 mm or less with larger lesions. We found that very small lesions were much less likely to be melanoma than larger lesions, meaning that many excisions in this size range resulted in benign diagnoses. However, some small lesions were melanoma, and a proportion were invasive, although they generally showed more favorable pathological features than larger melanomas. These findings suggest that lesion size can help clinicians refine decision-making, but it should not be used alone. A careful, risk-adapted approach may reduce unnecessary excisions while preserving early detection of clinically important melanoma.

## 1. Introduction

Cutaneous melanoma, although representing only a minority of skin tumors, accounts for more than 75% of skin-cancer-related mortality worldwide [1]. According to the most recent global cancer estimates, approximately 331,700 new melanoma cases and 58,700 deaths occur worldwide each year [1,2,3,4], with the incidence continuing to rise, especially in fair-skinned populations. UV exposure, particularly intermittent exposure and childhood sunburns, as well as phenotype, nevus count, and family history remain the strongest risk factors [5,6,7,8]. Early detection campaigns and diagnostic intensification, including dermoscopy, digital monitoring, and increased biopsy rates, have contributed to rising melanoma incidence [9,10,11]. However, mortality remained largely stable before the introduction of targeted and immune therapies, suggesting that a component of this rise may reflect overdiagnosis, identifying melanocytic lesions that would not have progressed to clinically significant disease [9,12,13].

Dermoscopy is considered the diagnostic gold standard for the evaluation of pigmented lesions and significantly improves sensitivity and specificity for melanoma compared with naked-eye examination [14,15,16,17,18]. It also improves the malignant-to-benign excision ratio—historically from ~1:18 to ~1:4.3 among trained users [19].

The diagnostic role of dermoscopy in triaging pigmented lesions is well established. Importantly, real-world evidence on dermoscopy-driven triage specifically in small lesions remains limited, and it is unclear whether lesions ≤ 6 mm selected for excision represent a subgroup with distinct clinicopathologic features, such as thinner tumors or a higher proportion of in situ disease [20].

Therefore, this retrospective cross-sectional study includes consecutive pigmented lesions referred for surgical excision with a preoperative diagnosis of melanoma based on clinical and dermoscopic assessment—i.e., lesions considered sufficiently suspicious to warrant excision, rather than routine removal of clinically banal nevi. In this setting, our focus is not the performance of individual dermoscopic criteria or algorithms but the real-world implications of a global dermoscopic impression of suspicion as used in everyday triage. We evaluated the diagnostic yield of excision for lesions ≤ 6 mm compared with larger lesions and described the clinical and histopathologic characteristics of melanomas diagnosed within these size strata. We used 6 mm as the main size threshold because this diameter is historically embedded in the ABCDE rule and is widely recognized in clinical melanoma assessment; nevertheless, this binary cut-off should be regarded as an analytic convention rather than a biologically meaningful boundary [20,21,22,23,24,25].

## 2. Methods

This retrospective cross-sectional study was conducted at a tertiary referral dermatologic oncology center within the Tuscany Regional Health Service in Florence, Italy. All pigmented lesions excised between January 2022 and December 2023 were screened. This study was conducted according to the Declaration of Helsinki and was approved by the local ethics committee.

We included consecutive pigmented lesions referred for excision with a preoperative diagnosis of melanoma or suspected melanoma based on clinical and dermoscopic assessment, rather than lesions removed for benign or cosmetic indications.

Clinical and dermoscopic assessment was performed in routine practice by dermatologists with experience in dermoscopy and melanoma diagnosis, including dermatologists working at the reference center and external dermatologists referring patients for surgical excision. The decision to excise was based on the overall clinicodermoscopic impression of melanoma or suspected melanoma. In equivocal cases, management could be discussed collegially according to routine departmental practice; however, this study did not include a predefined consensus reading protocol, blinded independent review, or formal assessment of interobserver agreement. Moreover, patients with a previous history of melanoma or high-risk features such as family history, genetic predisposition, immunosuppression, or atypical nevus syndrome were excluded, as were lesions excised without documented dermoscopic assessment and cases in which an incisional biopsy of the same lesion had previously been performed.

Cases that had undergone reflectance confocal microscopy before excision were excluded because the aim of this study was to evaluate diagnostic yield in lesions selected for excision on the basis of clinical and dermoscopic suspicion alone. This approach was chosen to reflect routine practice more accurately, given that reflectance confocal microscopy is not widely available across the territory.

Clinical and demographic data were extracted from institutional records, including age, sex, anatomical site, and clinical lesion diameter. Clinical lesion diameter was defined as the largest visible diameter of the lesion, measured in millimeters on clinical and dermoscopic photographs obtained at the time of excision. All images were acquired with a metric ruler placed adjacent to the lesion, allowing retrospective measurement from calibrated photographs.

Histopathologic diagnosis was recorded for all lesions, and in melanoma-confirmed cases, additional prognostic variables were collected: histologic subtype, Breslow thickness, ulceration, mitotic rate (mitoses/mm^2^), growth phase, presence of an associated nevus, regression, tumor-infiltrating lymphocytes, vascular or neural invasion, and microsatellitosis.

Data were anonymized before analysis. Descriptive statistics were generated for all variables, and associations between categorical variables were evaluated using the chi-square test or Fisher exact test, as appropriate. Statistical significance was set at *p* < 0.05 (two-sided). Analyses were performed using IBM SPSS Statistics, version 25 (IBM Corp., Armonk, NY, USA). In addition to univariate comparisons, a multivariable binary logistic regression model was fitted to identify factors independently associated with a histopathological diagnosis of melanoma (yes/no), including lesion diameter (>6 mm vs. ≤6 mm), age, sex, and anatomical site (back as reference). A sensitivity analysis modeled diameter as a continuous variable (per 1 mm increment). Model discrimination was assessed by the area under the ROC curve (AUC) and calibration by the Hosmer–Lemeshow test. These analyses were performed in R (version 4.x).

## 3. Results

A total of 2240 pigmented lesions were excised during the study period. The cohort included 50.9% female and 49.1% male patients, with a mean age of 53.2 years. The most frequently involved anatomical sites were the back (32.0%) and anterior trunk (25.1%), followed by the lower limbs (17.9%), upper limbs (12.2%), head and neck (7.3%), acral sites (3.9%), and mucosal areas (0.4%).

Overall, 609 melanomas were histologically confirmed, representing 27.2% of all excised lesions, corresponding to a malignant-to-benign ratio of 1:3.7. Among these melanomas, 51.4% were classified as melanoma in situ, and 48.6% were classified as invasive melanoma.

When stratified by lesion diameter, 1331 lesions (59.4%) measured ≤6 mm, and 909 lesions (40.6%) measured >6 mm.

The diagnostic yield differed significantly between these two categories: 175 melanomas (13.1%) were identified among lesions ≤ 6 mm, yielding approximately 1 melanoma per 7.6 excisions, whereas lesions > 6 mm demonstrated a markedly higher diagnostic yield, with 434 melanomas identified (approximately 1 melanoma per 2.1 excisions; *p* < 0.001). We then investigated even smaller lesions and discovered that, within the subgroup of very small lesions (≤4 mm), 51 melanomas were diagnosed among 692 excised lesions, reflecting an even lower ratio of approximately 1:13.6. Notably, no nodular melanomas were detected in this subgroup. (Table 1).

Distinct histopathologic patterns were observed between melanomas ≤ 6 mm and those >6 mm. Among ≤6 mm melanomas, 68.6% were in situ, and invasive tumors exhibited a mean Breslow thickness of 0.4 mm, with no cases of ulceration, mitotic activity present in only 13% of lesions, and vertical growth observed in 52.7%.

In contrast, melanomas > 6 mm were invasive in 55.5% of cases, with a higher mean Breslow thickness of 1.18 mm, ulceration present in 13.3% of lesions, mitoses detected in 39%, and vertical growth phase observed in 70.2%. Differences in ulceration, mitotic activity, and growth phase between size groups were statistically significant (all *p* < 0.05; Table 2).

In a multivariable logistic regression adjusting for age, sex, and anatomical site, lesion diameter > 6 mm was the strongest independent predictor of melanoma (adjusted OR 4.77, 95% CI 3.81–5.98; *p* < 0.001). Older age was independently associated with melanoma (OR 1.05 per year, 95% CI 1.04–1.06; *p* < 0.001), whereas sex was not (female OR 0.81, 95% CI 0.64–1.01; *p* = 0.063). The model showed good discrimination (AUC 0.826, 95% CI 0.807–0.845) and adequate calibration (Hosmer–Lemeshow *p* = 0.19) (Table 3A). In a sensitivity analysis modeling diameter as a continuous variable, each additional millimeter was associated with a 28% increase in the odds of melanoma (OR 1.28, 95% CI 1.24–1.33; *p* < 0.001; AUC 0.846), which discriminated significantly better than the dichotomous model (DeLong *p* < 0.001) (Table 3B).

## 4. Discussion

The present study provides real-world evidence regarding the diagnostic yield of excisions performed for lesions selected for excision because melanoma was clinically and dermoscopically suspected in a tertiary referral dermatologic oncology center, with particular attention to lesion diameter. Our data show that lesion size substantially influences the diagnostic efficiency of excision-based triage in routine practice.

More than half of all excisions in our series were performed on lesions ≤ 6 mm (1331 lesions, 59.4%), yet only 13.1% of these lesions were confirmed as melanoma. Accordingly, among every 100 lesions ≤ 6 mm removed because of clinicodermoscopic suspicion, approximately 87 were benign on histopathology. By contrast, nearly half of lesions > 6 mm were malignant. These findings indicate that the positive predictive value of excision for suspected melanoma declines markedly in smaller lesions, with a corresponding increase in the benign excision burden. In this sense, lesion diameter appears to be a clinically relevant modifier of diagnostic yield.

**Figure 1:**
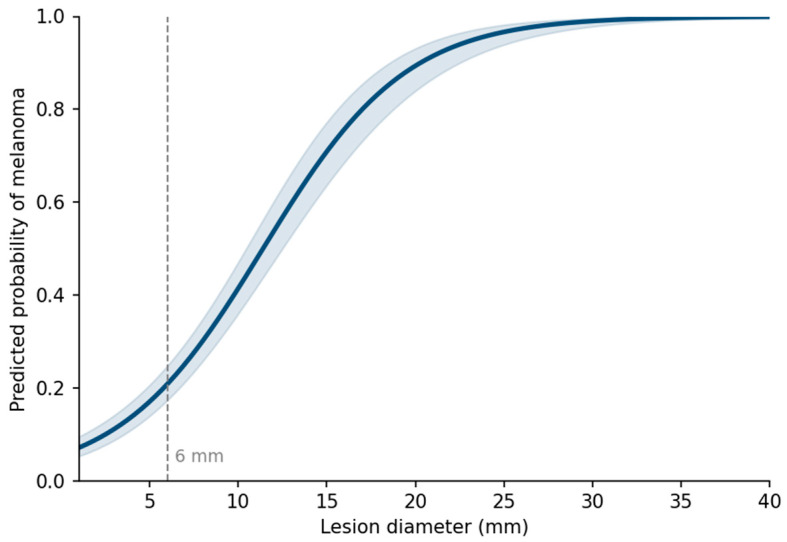
Predicted probability curve. Predicted probability of melanoma as a function of lesion diameter, derived from the multivariable logistic regression model with diameter as a continuous variable (adjusted for age, sex, and anatomical site; covariates set at mean age, male sex, and back as reference site). The solid line represents the predicted probability, and the shaded area represents the 95% confidence interval. The dashed vertical line marks the 6 mm threshold.

The multivariable analysis reinforces the central finding of this study. After adjustment for age, sex, and anatomical site, lesion diameter remained strongly and independently associated with melanoma diagnosis. Lesions > 6 mm had approximately 4.8-fold higher odds of being histopathologically confirmed as melanoma compared with lesions ≤ 6 mm. Importantly, this association was also confirmed in the sensitivity analysis treating diameter as a continuous variable, in which each additional millimeter in lesion diameter was associated with a significant increase in the odds of melanoma. This finding supports the presence of a graded relationship between lesion size and melanoma probability, rather than an effect limited to a single binary threshold (Figure 1).

These results suggest that the lower diagnostic yield observed in very small lesions was not simply explained by differences in patient age, sex distribution, or anatomical site but was independently related to lesion size. At the same time, these findings should be interpreted with caution. The study population consisted only of lesions already selected for excision because melanoma was clinically and dermoscopically suspected; therefore, the model estimates predictors of melanoma among excised suspicious lesions, not melanoma risk in the general population of pigmented lesions.

Overall, the adjusted analysis supports the concept that diameter is a clinically relevant modifier of diagnostic efficiency in dermoscopy-based triage. However, diameter should not be used as an isolated decision rule, and the 6 mm cut-off should be interpreted as an analytic convention rather than as a biologically meaningful boundary. Rather, lesion size should be integrated with dermoscopic morphology, lesion evolution, nodular architecture, anatomical site, and patient-level risk factors when calibrating the threshold for excision, particularly in small equivocal lesions.

Importantly, our findings should be interpreted as evidence of lower diagnostic efficiency in small lesions, not as evidence that small suspicious lesions should routinely be monitored rather than excised. A meaningful proportion of lesions ≤ 6 mm were melanoma, and a substantial subset of these were invasive. Therefore, lesion size alone cannot be used to determine management. Rather, diameter should be integrated with dermoscopic morphology, nodular architecture, anatomic site, documented evolution, and patient-level melanoma risk when calibrating the threshold for excision.

International epidemiologic studies have increasingly raised concerns regarding melanoma overdiagnosis, particularly involving thin and in situ lesions [9,10,11,12,13]. In high-screening environments, the incidence has risen substantially without a parallel reduction in mortality, suggesting that at least part of this increase may reflect the detection of biologically indolent disease [9,10,11,12,13]. Our results are consistent with this broader concern, insofar as the decline in diagnostic yield in progressively smaller lesions indicates that lowering the excision threshold is associated with a greater burden of benign excisions and, potentially, overtreatment. At the same time, our data do not allow us to determine which excisions were avoidable or whether surveillance would have been a safe alternative in individual cases. This distinction is important and should temper any interpretation of our findings in management terms.

Consistent with prior literature, melanomas diagnosed in lesions ≤ 6 mm showed a more favorable histopathologic profile than those arising in larger lesions [22]. In our cohort, approximately two-thirds of melanomas ≤ 6 mm were in situ, while invasive tumors in this subgroup had a mean Breslow thickness of 0.4 mm and no ulceration. In addition, invasive melanomas ≤ 6 mm demonstrated significantly fewer adverse histopathologic features, including mitotic activity and vertical growth phase, than melanomas > 6 mm. These data suggest that many melanomas detected in very small lesions are biologically earlier-stage tumors; however, they should not be interpreted as evidence that all such lesions are clinically trivial. Indeed, the presence of invasive melanomas within the ≤6 mm category argues against any rigid size-based rule for deferring biopsy. The exploratory analysis of even smaller lesions further supports the relevance of diameter in risk stratification. Within the subgroup of lesions ≤4 mm, only 51 melanomas were diagnosed among 692 excised lesions, corresponding to an NNE of 13.6, and no nodular melanomas were identified. This finding suggests that the benign excision burden may be concentrated particularly in the very-small-lesion range. Nevertheless, this subgroup analysis should be interpreted cautiously and should not be taken to imply that lesions below a fixed diameter threshold can be considered safe to ignore.

Historically, the ABCDE rule emphasized diameter > 6 mm as a suspicious feature [7]. With the increasing integration of dermoscopy into routine practice, clinicians are now more likely to identify and biopsy subtle abnormalities in very small pigmented lesions that might previously have been monitored or left unrecognized.

Welch et al. have argued that the progressive lowering of biopsy thresholds in the dermoscopy era may contribute to melanoma overdiagnosis and overtreatment and have questioned whether very small pigmented lesions (≤6 mm) should so readily trigger biopsy [9,10,11,12,13,21]. Our findings are aligned with this broader concern but do not support a rigid return to a purely size-based biopsy threshold. Rather, they support the view that lesion diameter is an important component of risk stratification: as lesion size decreases, the probability that an excised lesion will prove to be melanoma declines substantially, increasing the burden of benign excisions.

From a clinical perspective, the implications of these findings are nuanced. Excisional biopsy remains the reference standard when melanoma is clinically suspected. Suspicious nodular lesions, in particular, should be excised promptly regardless of size. By contrast, sequential digital dermoscopic monitoring may be considered for selected equivocal, non-nodular lesions, especially when overall melanoma risk is low and immediate histopathologic confirmation is not strongly indicated [26,27]. In this setting, lesion diameter may help refine management decisions but only as part of a broader clinicodermoscopic and patient-level risk assessment.

**Figure 2:**
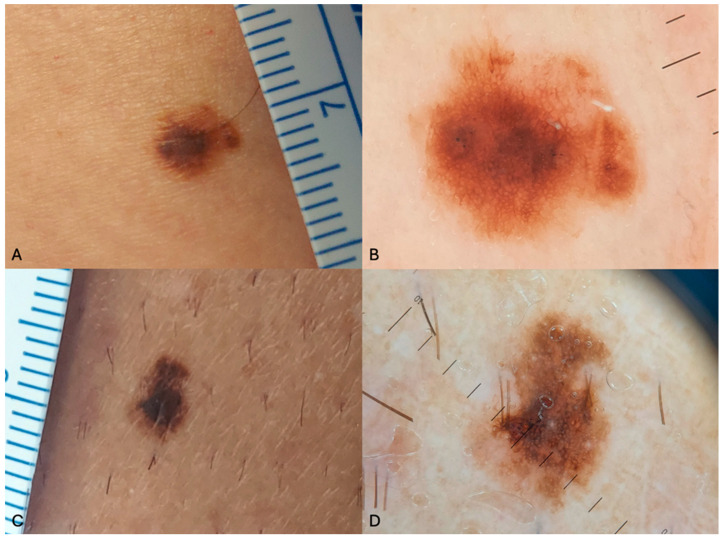
Clinical and dermoscopic images of 5 mm pigmented lesions excised for suspected melanoma. Case 1: (**A**) Clinical image of a 5 mm pigmented lesion on the right thigh of a 59-year-old woman. (**B**) Dermoscopy shows an atypical network with irregular globules. Histopathology confirmed melanoma in situ. Case 2: (**C**) Clinical image of a 5 mm pigmented lesion on the right lower leg of a 62-year-old man. (**D**) Dermoscopy shows an atypical network. Histopathology confirmed a lentiginous melanocytic nevus with cytologic atypia.

Over time, widespread dermoscopy use has lowered the perceptual threshold at which small lesions trigger concern, particularly in settings where clinicians face a high nevus burden and growing medico-legal pressure. Our data suggest that this downward shift of thresholds may come at the cost of a substantially reduced diagnostic efficiency. While dermoscopy is unquestionably valuable, improving accuracy and malignant-to-benign ratios [15,16,17], its performance in the subset of “micro-lesions” in routine practice remains suboptimal, and pattern-based dermoscopic criteria may be more difficult to interpret at that scale. Our findings should not be interpreted as dermoscopy “failing” in small lesions; rather, they suggest that the positive predictive value of a dermoscopic impression prompting excision is lower in lesions <6 mm. A likely explanation is that early or very small melanomas may display only subtle, focal, or incomplete dermoscopic features, increasing overlap with benign melanocytic lesions and reducing specificity (Figure 2).

Although the analysis of specific dermoscopic features leading to excision of very small pigmented lesions would be clinically valuable, this was beyond the scope of the present study. Individual dermoscopic criteria were not prospectively recorded using a standardized checklist, and images were not reassessed through a blinded central review. Therefore, our findings should be interpreted as reflecting the diagnostic yield of a global clinicodermoscopic impression of suspicion in routine practice, rather than the performance of specific dermoscopic structures or algorithms.

An important area for future research will be to establish a graded dermoscopic framework for small pigmented lesions, clarifying the relative diagnostic weight of individual features and defining which, and how many, classic dermoscopic criteria are required to substantiate clinical suspicion of melanoma.

An additional consideration is that excision is not a neutral act. Beyond the surgical procedure itself, benign excisions carry cosmetic consequences, healthcare-system costs, and potential psychological burden, particularly when a lesion is labeled or managed as a possible melanoma [28,29,30]. For this reason, efforts to optimize specificity are clinically relevant, provided that they do not compromise the timely diagnosis of clinically significant melanoma. Our data suggest that this balance becomes especially important in the very small lesion range, where the benign excision burden is highest.

This study has several limitations. It is retrospective, is single-center, and was conducted in a high-expertise dermoscopy setting, which may limit generalizability. Another limitation concerns interobserver variability. Dermoscopic assessment and biopsy decisions were performed in routine clinical practice and were not standardized through a predefined scoring system, central image review, or blinded assessment by multiple independent observers. Consequently, we could not calculate interobserver agreement or determine whether the threshold for excision varied among clinicians. This is particularly relevant for small pigmented lesions, in which dermoscopic criteria may be subtle, focal, or incomplete. Nevertheless, this limitation also reflects the real-world nature of this study, whose objective was to estimate the diagnostic yield of lesions selected for excision in everyday dermoscopy-based triage rather than to validate the reproducibility of individual dermoscopic criterion.

Dermoscopic assessment and biopsy decisions were not standardized across clinicians. Most importantly, because only excised lesions were included and non-excised lesions were not prospectively monitored, this study cannot determine whether alternative management strategies, such as surveillance, would have been safe or cost-effective. In addition, the exclusion of patients with a previous history of melanoma limits the applicability of our findings to higher-risk populations.

A key strength of this study is the large consecutive real-world series of lesions excised because melanoma was clinically and dermoscopically suspected. Importantly, the analysis includes a substantial subgroup of very small lesions (≤6 mm), allowing a robust estimate of diagnostic yield in a clinically challenging setting. Rather than supporting size alone as a management rule, the marked gradient in NNE across diameter categories provides practical evidence that lesion size materially affects the efficiency of excision-based triage.

## 5. Conclusions

In conclusion, our findings support the view that lesion diameter is an important risk-stratification variable in melanoma triage. As lesion size decreases, diagnostic yield falls, and the burden of benign excisions rises, increasing the risk of overtreatment if biopsy thresholds are lowered indiscriminately. However, because melanomas, including invasive melanomas, also occur in lesions ≤ 6 mm, diameter should modify rather than replace clinicodermoscopic judgment. A more selective, risk-adapted dermoscopic approach to very small equivocal lesions may help improve specificity, while preserving prompt excision for lesions with convincing melanoma-specific features.

## Figures and Tables

**Table 1:** Frequency and proportion of melanomas among excised pigmented lesions across clinical diameter categories.

Diameter	Melanoma (*n*)	Non-Melanoma (*n*)	Total Excised	Melanoma (%)	Melanoma-To-Excision Ratio
1 mm	0	15	15	0.0%	—
2 mm	1	101	102	1.0%	1:102.0
3 mm	12	198	210	5.7%	1:17.5
4 mm	38	327	365	10.4%	1:9.6
5 mm	53	281	334	15.9%	1:6.3
6 mm	71	234	305	23.3%	1:4.3
7 mm	76	175	251	30.3%	1:3.3
8 mm	57	102	159	35.8%	1:2.8
9 mm	52	72	124	41.9%	1:2.4
10 mm	39	41	80	48.8%	1:2.1
11 mm	25	21	46	54.3%	1:1.8
12 mm	32	23	55	58.2%	1:1.7
13 mm	34	11	45	75.6%	1:1.3
14 mm	21	7	28	75.0%	1:1.3
≥15 mm	94	27	121	77.7%	1:1.3

**Table 2:** Histopathologic features of invasive melanomas ≤ 6 mm and >6 mm in diameter. The analysis includes only invasive melanomas. Values are counts and percentages; odds ratios derived from 2 × 2 comparisons.

Characteristic	≤6 mm Melanomas *n* (%)	>6 mm Melanomas *n* (%)	*p*-Value	Odds Ratio (95% CI)
Ulceration present	0 (0.0)	32 (13.3)	**0.0088**	**17.22 (1.04–285.65)**
Mitoses present	7 (12.7)	94 (39.0)	**0.0004**	**4.38 (1.90–10.10)**
Regression present	15 (8.6)	47 (10.8)	0.4928	1.30 (0.70–2.38)
Associated nevus present	16 (29.1)	60 (24.9)	0.6373	0.81 (0.42–1.55)
Vertical growth phase	29 (52.7)	167 (70.2)	**0.0204**	**2.11 (1.16–3.83)**
Radial growth phase	26 (47.3)	71 (29.8)	—	—
Vascular invasion present	0 (0.0)	6 (2.5)	0.5082	3.10 (0.17–55.91)
Neurotropism present	0 (0.0)	5 (2.1)	0.6124	2.61 (0.14–47.99)
Microsatellitosis present	0 (0.0)	4 (1.7)	0.7464	2.13 (0.11–40.15)

**Table 3:** Multivariable logistic regression. (A) Multivariable logistic regression—dichotomous diameter. (B) Multivariable logistic regression—continuous diameter (sensitivity analysis).

**(A)**			
**Predictor**	**OR**	**95% CI**	***p***
**Diameter > 6 mm (vs. ≤6 mm)**	**4.77**	**3.81–5.98**	**<0.001**
Age (per year)	1.05	1.04–1.06	<0.001
Age (per decade, +10 years)	1.63	1.51–1.74	<0.001
Female sex (vs male)	0.81	0.64–1.01	0.063
Site: anterior trunk (vs back)	0.68	0.51–0.91	0.010
Site: lower limbs (vs back)	1.33	0.97–1.82	0.082
**Site: upper limbs (vs. back)**	**1.68**	**1.19–2.38**	**0.003**
Site: head and neck (vs back)	0.56	0.36–0.87	0.011
**Site: acral/mucosal (vs. back)**	**0.13**	**0.03–0.37**	**<0.001**
**(B)**			
**Predictor**	**OR**	**95% CI**	***p***
**Diameter (per +1 mm)**	**1.28**	**1.24–1.33**	**<0.001**
Age (per year)	1.04	1.04–1.05	<0.001
Female sex (vs. male)	0.88	0.70–1.11	0.286
Site: anterior trunk (vs. back)	0.68	0.50–0.92	0.012
Site: lower limbs (vs. back)	1.34	0.97–1.85	0.075
**Site: upper limbs (vs. back)**	**1.71**	**1.19–2.44**	**0.003**
Site: head and neck (vs. back)	0.51	0.31–0.81	0.005
**Site: acral/mucosal (vs. back)**	**0.13**	**0.03–0.38**	**0.001**

## Data Availability

The data that support the findings of this study are available from the corresponding author upon reasonable request.
